# Thalamocortical loops as temporal demodulators across senses

**DOI:** 10.1038/s42003-023-04881-4

**Published:** 2023-05-26

**Authors:** Ehud Ahissar, Guy Nelinger, Eldad Assa, Ofer Karp, Inbar Saraf-Sinik

**Affiliations:** Department of Brain Sciences, Weizmann Institute, Rehovot, 76100 Israel

**Keywords:** Thalamus, Sensory processing, Thalamus, Thalamus, Whisker system

## Abstract

Sensory information is coded in space and in time. The organization of neuronal activity in space maintains straightforward relationships with the spatial organization of the perceived environment. In contrast, the temporal organization of neuronal activity is not trivially related to external features due to sensor motion. Still, the temporal organization shares similar principles across sensory modalities. Likewise, thalamocortical circuits exhibit common features across senses. Focusing on touch, vision, and audition, we review their shared coding principles and suggest that thalamocortical systems include circuits that allow analogous recoding mechanisms in all three senses. These thalamocortical circuits constitute oscillations-based phase-locked loops, that translate temporally-coded sensory information to rate-coded cortical signals, signals that can integrate information across sensory and motor modalities. The loop also allows predictive locking to the onset of future modulations of the sensory signal. The paper thus suggests a theoretical framework in which a common thalamocortical mechanism implements temporal demodulation across senses.

## Abstract (web summary)

A theoretical framework proposes that a common thalamocortical mechanism implements temporal demodulation across senses.

## Introduction

Brains are made of loops. Arguably, no open-loop (i.e., open ended, or feedforward only) pathway can be found in the brain (sensory organs included). Among these loops, thalamocortical (TC) loops have received specific attention. Their anatomical pattern seems to be similar across modalities^1^, and implies a tight linkage between the activity of thalamic and cortical neurons that process the same sensory or motor information^2–13^. Do these anatomical similarities imply a similar function? And what kind of function can be implemented by such tight thalamocortical anatomical linkage?

Over the years, TC loops have been proposed to implement various functions, from tuned oscillators^14^ to attentional filters^15,16^. Of the various proposed functions, three have gained significant empirical support. The first is that thalamic neurons function as switchable, or tunable, relay stations. Namely, they relay (i.e., amplify, clean, and replicate their input without changing its content or coding scheme) afferent information, with the cortical feedback tuning their relay operation, or even switching it entirely on or off^17^. The second proposal also asserts that the thalamo-cortical feedforward connections relay afferent information. The cortico-thalamic feedback, however, includes connections that are part of downstream processing, relaying cortical outputs to other cortical circuits via thalamic pathways^16,18^. The third proposal is that TC loops function as neuronal phase-locked loops (NPLLs), which recode temporally encoded afferent signals in population firing rates^19,20^.

The latter proposal is especially relevant in perception, where the scanning motion of the sensor interacting with the external world generates the sensory information brains can process^21^. Converting the spatial characteristics of objects into temporal structures of the activity of receptors (i.e., temporal encoding) gives temporal processing a major role in natural perception^22^. We thus review how NPLLs can implement recoding of temporally encoded sensory information in three modalities: touch, vision and audition. The article describes the nature of sensory temporal encoding in these three modalities, and the principles proposed for central recoding in each of them. Importantly, while the principles of time-to-rate recoding are proposed to be similar across the three sensory modalities, the parametric regimes are expected to be modality-specific. The article thus presents the possibility that mammalian thalamocortical systems translate temporal-coding to rate-coding via modality-specific neuronal phase-locked loops. It reviews the empirical support for this possibility, describes its theoretical advantages and outlines relevant empirical predictions. As the empirical evidence supporting the operation of the NPLL model across senses is partial, and not balanced across senses, the current paper should be taken as a proposal of a theoretical framework that calls for direct testing rather than as a summary of well-supported ideas.

## Temporal encoding

### Temporal encoding induced by sensor motion

The scanning motion by the sensory organ transforms the spatial structure of external features into the temporal domain. The scanning direction dictates the activation order of individual and neighboring receptors. During the continuous scanning motion, a straightforward formula (1) describes this spatio-temporal transformation, mapping spatial distances (*dx*) into temporal delays (*dt*, between sequential neuronal activations), depending on the scanning velocity of the sensory organ (*v*)^19,20,23^:

1 $$\,{dt}=\frac{{dx}}{v}.$$

With this transformation, spatially closer features induce shorter activation delays. Importantly, since the values of these temporal delays depend on the scanning velocity, which is under the active control of the animal, temporal encoding, such as in mammalian touch and vision, allows hyperacuity. Slowing down the scanning motion effectively magnifies the finer spatial details of the external object. Thus, fine resolution is no longer limited by the granularity of the sensory receptors (e.g., photoreceptors at the retina or mechanoreceptors at the fingertip^22^). Instead, a satisfactory resolution is dictated by the accuracy of motor control and its compatibility with the temporal sensitivity of the sensory receptors and their downstream processing^24–26^. Of course, since the sensory-motor compatibility also dictates the range of sensory signals the system can acquire, the specific temporal delays, scanning velocities and resulting spatial resolutions are likely to differ between modalities, and also between species for the same modality.

Active control of the scanning velocity, in a way that is adaptive to the resolution requirements (that dynamically change during behavior) and to the specific features of the object in focus, can be achieved via motor-sensory-motor (MSM) closed loops^27^. MSM loops actively modulate key parameters of the scanning motion in a dynamically adaptive way that allows, for example, decreasing the scanning velocity when encountering a fine-textured object or when a higher resolution is behaviorally meaningful. In addition, MSM loops introduce the information about sensor motion inherently into the spatio-temporal computation, allowing the distinction between self-motion and external movements.

### Temporal encoding induced by stimulus dynamics

Whereas the mammalian tactile and visual receptor-arrays sense transient changes in both the spatial and temporal structure of external energies, auditory receptor-arrays sense transients only in the temporal structure of external energies. Accordingly, while in touch and vision temporal predictions (e.g., predictions of activation onset times) can refer to the interactions between sensor motion and external spatial details, in audition they refer exclusively to the temporal structure of the auditory stimulus itself^27,28^.

In speech, a prominent temporal patterning of the signal is carried by the temporal envelope – the signal describing the changes in the amplitude of sound over time - that reflects its syllabic structure and typically occurs at frequencies below 8 Hz^29^. Parsing the speech signal according to its temporal envelope is considered a necessary step in auditory processing^30^. The efficiency of such parsing, which affects processing resolution, is limited in two aspects. First, the processing bandwidth of the human auditory system limits the comprehensible syllabic frequency^30–32^, and second, the accuracy of phase-locking in the auditory system likely limits its ability to decipher syllabic onsets, hence limiting sentence-by-sentence intelligibility^28,31,33,34^.

## Central recoding

Why should temporally encoded signals require recoding? There are two major possible reasons. One, such recoding may allow integration with neuronal information that is coded differently. Second, recoding may be required to allow the integration of the temporally encoded information in the dynamics of MSM loops. We hypothesize that the common coding scheme, which allows integration across sensory and motor modalities, is rate coding^20,35^.

**Fig. 1:**
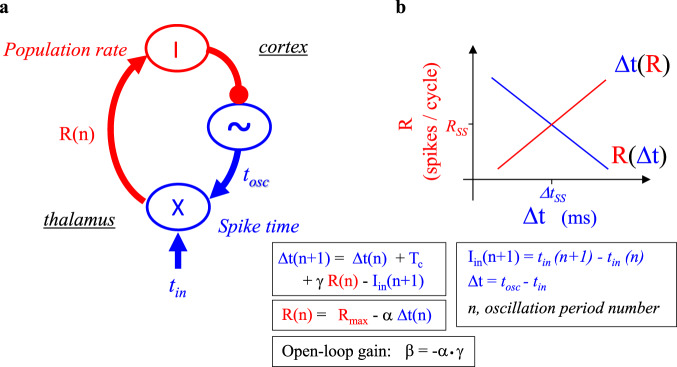
Spike-time to population-rate transformation by TC NPLL. **a** Schematic description. Temporal variables are in blue and rate variables in red. *t*, time; R, rate; n, cycle number; X, comparator; ~, rate-controlled oscillator (RCO); T_c_, the intrinsic period of the RCO; R_max_, maximal value of R; I, pool of inhibitory units. **b** Phase-plane description. The two transfer functions describe the reciprocal relationships between the temporal (Δt) and rate (R) variables, leading to a fixed point at their intersection. This fixed point ([Δ*t*_ss_, *R*_ss_]) is the steady-state the loop tries to converge to. The equations describe a linear NPLL; the behavior of non-linear NPLLs can be assessed near the fixed point using linear approximations (see refs. ^19,20,23^).

An efficient, arguably the most efficient^36^, circuit for temporal-to-rate transformation is the neuronal phase-locked loop (NPLL)^20,37,38^. The NPLL uses a local rate-controlled oscillator (RCO) that serves as an adaptive predictor for the temporal structure of the loop’s incoming signals. Any deviation of the incoming spike times from the RCO’s predicted times is informative, and is recoded as a rate-coded signal (Fig. 1). Temporal modulations in the NPLL’s input signal are thus instantaneously converted to rate modulations in its output signal; neither the input nor the output are required to be periodic for that to happen.

The NPLL has two arcs (Fig. 1a), each performing an opposite transformation: the bottom-up arc (red), transforms temporal delays into rate code (*Δt → R*), and the top-down arc (blue), transforms population rates to temporal delays (*R → Δt*). To intuitively explain how the NPLL functions, we follow the information flow step-by-step. The temporally encoded signal comes in the bottom-up direction, conveying information from the sensory receptors. The NPLL compares the incoming firing times (*t*_*in*_) with the firing times of its cortical oscillator (*t*_*osc*_). Acting collectively as an AND gate, the population of thalamo-cortical neurons fire only when the bottom-up and top-down signals overlap. This AND gating generates a population rate that is inversely proportional to the delay between the bottom-up and top-down signals, with high firing rates corresponding to smaller delays. Next, this integrated rate-coded signal drives a population of inhibitory neurons that closes the loop projecting onto the cortical oscillator. Functionally, the negative feedback leads the convergence of the loop: since the population rate is a decreasing function of the temporal delay, whereas the latter is an increasing function of the former (Fig. 1b), the loop will converge towards a single stable fixed point defined as a pair of values (*R*_*SS*_ and *Δt*_*SS*_; intersection in Fig. 1b). Importantly, this fixed point is determined anew for every new input spike, thus forcing the cortical oscillator to track the timing of the input. The cycle-by-cycle convergence dynamics is determined by the transformation parameters in both arcs. The combined gain of the two arcs, β (Fig. 1), defined as the “open-loop gain”, expresses how fast the loop locks onto the input signal. The larger the magnitude of β, the faster the convergence and the lower the stability of the loop^20,39^.

It should be emphasized that the oscillator (RCO) of the NPLL may be implemented in many ways, not necessarily in the form of single-cell sustained oscillators. Any form of local oscillations, whose frequencies are tunable locally within the range that is relevant to the expected sensory frequencies, and that are effectively expressed during active perception, will satisfy the requirements of NPLL.

### Touch

Tactile information is collected by skin mechanoreceptors and, in rodents, also by vibrissal (whiskers’) mechanoreceptors. The sensation in primates and rodents is primarily active, achieved by scanning objects of interest with the relevant sensory organs in a manner that adapts to the tactile features of the object^24,40–46^. Consequently, fine spatial details representing shapes and textures are likely encoded temporally by the mechanoreceptors populating the moving sensor^20,22,24,25,44,47^. NPLLs would make excellent re-coders of this information, recoding the information in population firing rates. Is there evidence for NPLLs operating in tactile systems?

In primates, it was found that local cortical oscillators in the second somatosensory cortex (S2) lose their periodicity when the monkey touches objects^19^. This observation is consistent with these oscillators functioning as part of an NPLL circuit, because the NPLLs’ oscillators are forced to track the instantaneous periodicity of the input. The periodicity of the input is determined by the interactions between hand motion and the object’s spatial frequencies (see Eq. 1). Periodic tactile inputs thus can be generated only when the instantaneous hand speed is inversely correlated with the local spatial frequency of the object’s surface texture, a correlation that typically does not occur. These observations are consistent with an NPLL scheme, but cannot discriminate between NPLL and other possible processing schemes. For such a discrimination, a more systematic testing is required.

Such a systematic study, aimed at testing NPLL predictions, was conducted in rodents. The major predictions tested were (i) that somatosensory cortical oscillators can track the frequency of periodic tactile stimulations and (ii) that the phase difference between the tactile input and cortical oscillations increases with the frequency^20^. The latter prediction distinguishes NPLLs from coupled oscillators^37^. Both predictions were confirmed in rodents^22,39,48–50^. Importantly, for frequencies within the range of active whisking (~5–11 Hz), the latency prediction (prediction ii) was confirmed only for neurons belonging to the paralemniscal pathway (in the medial division of the posterior nucleus of the thalamus, POm, and in layers 5 A and 2/3 of the primary somatosensory cortex, S1).

**Fig. 2:**
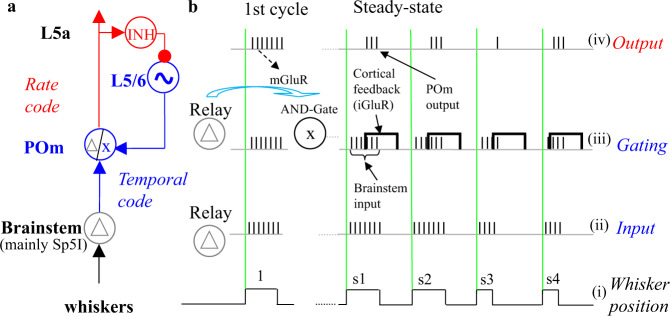
Implementation of a tactile NPLL in a vibrissal, paralemniscal, TC loop. **a** The basic phase-locked loop. POm neurons, exhibiting two functional modes (Δ, relay mode; x, AND-gate mode), drive cortical inhibitory neurons (INH), which in turn inhibit cortical oscillatory neurons (~) that drive the cortical feedback. •, inhibitory connections; ⇾, excitatory connections. **b** A timing diagram describing the transition of POm neurons from relay (Δ) to AND-gate (x) mode, and the effect of the stimulus pulse-width. Short black vertical lines represent spikes. Green vertical lines represent stimulus-pulse onsets. Traces: (i) whisker deflection, up denotes protraction. (ii) brainstem response. (iii) AND-gate operation at the POm: bold rectangles represent the delayed cortical feedback; only those brainstem spikes overlapping with the cortical feedback would “pass the gate”. Spikes are plotted with a short delay after the brainstem spikes. (iv) the output of the POm. Following a quiescent period, POm neurons are hyperpolarized and thus shift into a relay mode. The response to the first stimulus cycle (cycle 1) is relayed to the cortex (activity not shown in the diagram). The cortical feedback activates metabotropic (mGluR) and ionotropic (iGluR) receptors at the thalamus. The slow mGluR activation depolarizes the thalamic neurons and shifts them into an AND-gate mode, in which brainstem activity will “pass the POm gate” only when additional cortical feedback (via iGluR) is active. When the stimulus pulse-width is shortened (cycles s3 and s4), the latency of the cortical feedback has to decrease in order to keep the output spike-count constant (in order to maintain the RCO frequency matching the input frequency, which remained unchanged). Adapted from^134^.

**Fig. 3:**
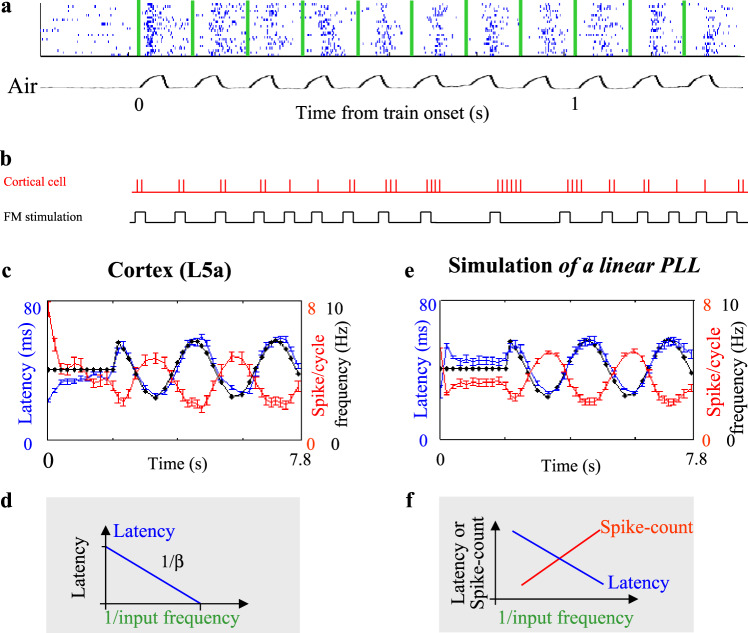
Working regime of paralemniscal NPLLs. **a** Example of L5A responses to constant frequency (CF) air-puff stimulations of the whiskers (blue, raster; green, air-puff onset time; black, air pressure temporal profile). Adapted from ref. ^48^. **b** Schematic description of a frequency modulated (FM) stimulation and the corresponding response of a cortical cell. **c** L5A responses to CF (2 s) followed by FM (5.8 s) stimulation. Response latency (blue) and spike rate (red) are plotted with the instantaneous frequency (1/inter-stim-interval; black). Errorbars denote SEM. **d** A graph describing the relationships between latency and the input frequency at steady-state, from which the open-loop gain, β, can be computed if the circuit functions as an NPLL^19,20,23^ (see equations in Fig. 1). **e** Simulation of NPLL with the open-loop gain extracted from the experiment depicted in c (*β* = −5.4). **f** The trends of the steady-state dependencies of latency and spike count on the input frequency as predicted in NPLLs.

These results suggest that at least some paralemniscal thalamocortical loops, between POm and layer 5/6 of S1, function as NPLLs^51^. For these circuits, the accumulating evidence suggest that (i) TC circuits could switch between relay and NPLL modes, by switching the thalamic neurons between relay and gate modes, respectively, under cortical control (Fig. 2); (ii) if NPLLs are indeed part of TC loops, they may function in a regime of large open-loop gains (|β| > 2; Fig. 3), entailing unstable but fast-reacting loops;^20,37^ (iii) the TC loops that can function as NPLLs are composed of specific ensembles of POm and S1 neurons;^52,53^ and (iv) TC loops connecting POm with S2 can also function as NPLLs^54,55^.

NPLLs convert spike-time information to rate-coded signals. If NPLLs are implemented in thalamocortical loops, then thalamic and cortical neurons operating within the NPLL loop are expected to code sensory information in both time and rate (Figs. 1 and 2), whereas the firing of downstream cortical neurons is expected to be dominated by rate-coding. This is indeed the case with primates performing tactile frequency discrimination tasks^56^ – the temporal information of the two stimuli is preserved well in thalamic and S1 neurons and fades away in downstream stations. In parallel, downstream stations are able to perform computations that are based on these rate-coded signals. Furthermore, consistent with the population rate-code used in feasible implementations of thalamocortical NPLLs^20^ (Fig. 1), it was found that population rate-coded signals are more reliable than single-cell rate-coded signals in processing and comparing the frequencies of the sensory signals^56^. The observed population rate code obeys the prediction of the NPLL, as it is based on a monotonously changing rate as a function of the sensory frequency^56^ (Figs. 1 and 3).

### Vision

Visual information is collected by photoreceptors populating the retina of constantly moving eyes^57–59^. As with active touch, active vision induces temporal coding, by which spatial offsets are encoded by temporal delays^22^. Importantly, reliable coding of fine spatial details occurs only with temporal coding. This is because (i) retinal temporal coding is significantly more precise than retinal rate coding^60,61^, and (ii) the continuous motion of the eyes significantly contaminates rate coding, for which integration over a certain time-window is required. Given that the spatial scanning amplitude during a typical fixational pause (the period between two successive saccades) is 2–3 orders of magnitude larger than the finest perceivable spatial offset^22,58,62^, temporal coding appears more compatible with the observed perceptual acuity than spatial coding.

**Fig. 4:**
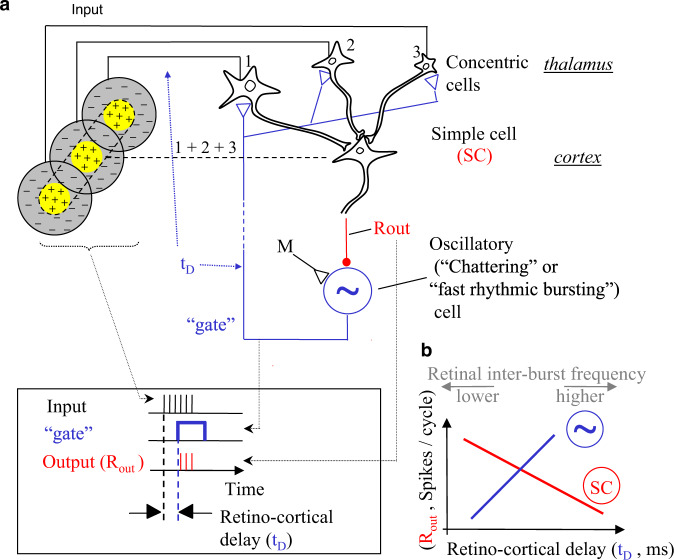
Implementation of a visual NPLL by a thalamocortical loop. **a** A schematic description of the proposed thalamocortical closed-loop decoder. The scheme is based on the schematic description of the feedforward connectivity suggested by Hubel and Wiesel^63^; the feedback connectivity added in blue closes the loop in a way that permits a PLL-like operation. Excitatory and inhibitory connections are represented by open triangles (▷) and solid circles (●), respectively. Dashed line indicates possible poly-synaptic link. Input, retino-thalamic input; SC, simple cells; M, modulatory excitatory input; ~, oscillatory neurons. Inset: Implementation of the phase detection function by cortico-thalamic gating: the Output is active only when both the Input and the “gate” are active. **b** Schematic phase plane of the two basic transfer functions of the loop. SC’s transfer function (red): Output spike-count (*R*_out_) decreases as the retinocortical delay (*t*_D_) increases. Oscillatory cells transfer function (blue): *t*_D_ increases as *R*_out_ increases (note reversal of axes here). The crossing point of the transfer functions is the set point for a specific retinal temporal frequency. The gray arrows on top relate the direction of change of the inter-burst frequency of the retinal input to those of the phase-plane variables: directly related to *t*_D_ and inversely related to *R*_out_. Adapted from ref. ^62^.

When the eyes are stationary, cortical simple-cells typically fire strongest in response to bars that are oriented in parallel to their elongated axis and are moved across it^63^. The temporal encoding induced by eye movements, however, achieves its highest resolution by encoding along, rather than across, the elongated axes of simple-cell receptive fields^62,64^. This is a consequence of the anatomical structure of these receptive fields (Fig. 4a). In this coding scheme, the fine details of shape are encoded by inter-receptor temporal phases, texture by instantaneous intra-burst rates of individual receptors, and motion by inter-burst temporal frequencies. The visual system can read the encoded information in several ways. NPLLs offer significant advantages also here. Specifically, NPLLs can lock to the retinal jitter and thereby (i) recode external motion in population rates and (ii) set temporal windows for efficient processing of shape and texture by downstream circuits.

How could NPLLs be implemented in the visual system? Here too, thalamocortical loops appear naturally tuned to function as NPLLs (Fig. 4). All it takes is closing the loop from simple cells (SCs) to the thalamus via cortical oscillators. Note that the cortical oscillators can come in various forms. In principle, almost any neuron can function as a local oscillator with certain distributions of its ionic channels^65,66^. Visual cortical oscillations can function within NPLLs if their frequencies (i) are in the range of the input frequencies and (ii) can be modulated by local cortical inputs. Mammalian cortical oscillations fulfill both criteria. The spectral densities of human fixational eye movements (FeyeM) and neuronal oscillations in the visual cortex of monkeys^67^ exhibit a striking similarity; both emphasize alpha and gamma modes, see^22^. Oscillations at frequency ranges that match those of FeyeM were observed in the visual cortex of cats, ferrets and monkeys^67–72^. The frequencies of visual cortical oscillations can be controlled locally^69–71^ and can be modulated by and locked to external stimuli^71–74^, as is the case in other modalities.

Besides frequency ranges, the main difference between visual and other cortical oscillations is that single-cell visual oscillations are usually not observed in the absence of visual stimuli. This might indicate that the expression of cortical oscillations (e.g., translation of sub-threshold oscillations to spike activity) requires an additional excitatory input^69,71^. Such an input (‘M’ in Fig. 4) can be provided by an internal preparatory signal or an afferent stimulus-driven signal, and shaped during development and learning^75^. Indeed, evidence for preparatory enhancement of local oscillations in the visual cortex has been accumulating over the years^76–79^.

### Audition

Touch and vision share a key perceptual strategy: they scan their environments with a lateral motion of two-dimensional arrays of receptors. Since receptors are mostly sensitive to changes (within their dynamic ranges), sensor motion is what allows the perception of stationary objects, encoding space by time^22,80^. The transduction of auditory signals (i.e., pressure waves) to neural signals is performed in the cochlea where the basic organization of receptors is different than the one observed in touch and vision. Instead of the two-dimensional array of dynamically-similar receptors which support two-dimensional representation of space, audition is based on a uni-dimensional array of dynamically different receptors supporting a uni-dimensional representation of an auditory frequency spectrum.

As a result, auditory sensory activation is less dependent on self-motion, a fact that probably puts audition in a motor-sensory regime that is distinct from those of touch and vision. Thus, if hearing involves sensor motion, it is not to directly serve the encoding of spatial offsets. Instead, active hearing may involve the encoding of spectral offsets (along the basilar membrane) by time and the tuning of the basilar membrane to match acoustic expectations by controlling the tension of the outer haircells^81,82^. Such implementations of active sensing are of course different from the visual and tactile implementations. In vision and touch, efference control of the sensory organ is manifested as overt sensor motion. In audition, instead, efference control results in covert tiny movements of the outer haircells, which affect the way ongoing auditory signals are perceived.

Temporal modulations are therefore inherent components of acoustic signals, with or without active cochlear mechanisms. One such modulation, as mentioned above, is the temporal envelope of speech, which defines the slow variations of the spectral energy of a spoken sentence, variations that are usually below 8 Hz^29^. This low-frequency information is crucial for identifying phonemes, syllables, words, and sentences^83^. Indeed, speech comprehension depends on the integrity of its temporal envelope^84,85^. The mechanisms by which this information is extracted and processed are still unknown. We previously suggested that circuits that can facilitate the processing of the temporal envelope, and the locking of downstream processing of the spectral information to its phasic changes (indicating syllable onsets), are thalamocortical NPLLs^28,31^.

**Fig. 5:**
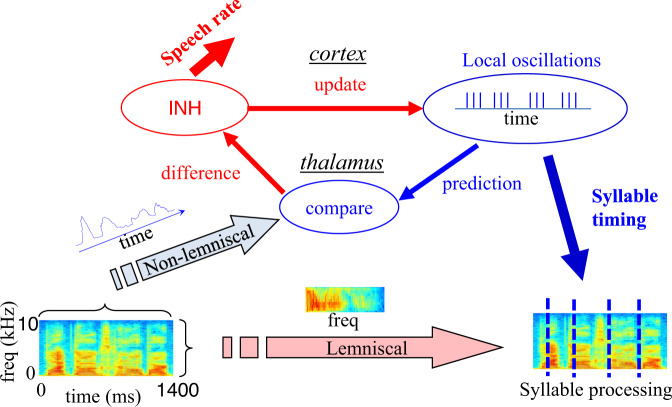
Possible roles of auditory TC NPLLs in the processing of the temporal envelope and in syllable processing. We postulate that the temporal envelope of the speech signal is processed in parallel to pre-processing of its spectral content, probably by the non-lemniscal and lemniscal systems, respectively. In this scheme, the temporal envelope is compared against a cortical reference (temporal expectation) implemented by local neuronal oscillations. The comparison occurs in the (non-lemniscal) thalamus, and its output (which is proportional to the temporal difference between the two signals) is fed-back to the cortex, where it eventually updates the reference signal (likely via inhibitory neurons, INH). The signal that represents the temporal difference, which contains information about input speech rate, might be further processed (red arrow, top left). Cortical oscillations, which express an expectation for the onset timing of the next syllable, send this timing information to a postulated “syllable processor,” which processes the spectral information contained in a syllable. This expectation is updated from syllable to syllable until stabilized on the speaker’s rhythm^27,28^. Spectograms are of the sentence ‘black dogs cannot bark’^31^.

How would such NPLLs work? In principle, many neuronal circuits could implement the PLL algorithm, including sub-cortical and cortico-cortical loops. Based on the findings in the tactile and visual systems, we speculate that auditory NPLLs are implemented in thalamocortical loops, and that a temporal comparison takes place in the thalamus, most likely in the non-lemniscal nuclei. As in the vibrissal tactile system, the auditory non-lemniscal thalamus exhibits larger temporal dispersion^86,87^ and spectral integration^87–89^ than the lemniscal thalamus. Both features, together with a typical thalamic gating mechanism^90,91^ and a strong cortico-thalamic feedback^92^, make the non-lemniscal thalamus optimal for temporal comparison functions^20,22,52^. According to this hypothesis, the non-lemniscal thalamus produces a difference signal that is fed back to the cortex, where it is used to update the frequency of the intrinsic oscillators. If the entire loop is connected as a negative feedback loop, via thalamic^93^ or cortical inhibition, the negative feedback would force the cortical oscillations to phase-lock to the envelope of the speech signal (Fig. 5).

The spectral analysis of syllables must be coordinated with syllable timing^94^. Our hypothesis suggests that the local oscillators of the NPLLs, which track the syllabic rate and thus predict the onset timing of the next syllable, trigger the spectral processing of each syllable. This predictive signaling by an adaptive internal clock can prevent losing the important spectral information contained close to the syllable onset. The existence of such intrinsic clock is also consistent with a phenomenon often related to in speech perception: that of perceptual center (P-center^95,96^). The P-center of a signal, such as a syllable, corresponds to its “psychological moment of occurrence”^95^. Thus, the experimental definition of a P-center of a speech signal depends on a comparison, by a listener, between such psychological moments of occurrence and an internal temporal ruler, or a pacemaker^95,97^. NPLLs exhibit such a process: comparing the rate of an input stream of syllables against an internal temporal ruler in the form of intrinsic oscillations.

Thus, the suggestion here is that speech processing includes at least two streams that eventually converge: those of the temporal envelope and the spectral content (Fig. 5). Supporting evidence for such a separation, in addition to the anatomy of afferent auditory pathways described above, can be found in the response selectivities of neuronal populations in the primary auditory cortex^98^ and in the anatomy of cortico-thalamic connections^99–101^. Supporting evidence for the oscillations-based processing of the temporal envelope comes from a series of studies that demonstrate the dependency of speech comprehension on frequency and phase matching between the temporal envelope and cortical oscillations^28,30–32,102,103^, as well as from theoretical work^104^.

### Comparison across the senses

Our senses collect complementary information about our environment and, thus, many of their aspects are necessarily different. Still, common principles can be found. Both touch and vision employ two-dimensional sheets of receptors (under the skin, around the whisker follicle, or on the retina) that are activated by temporal modulations. Since most of the sensed field is usually stationary, these temporal modulations primarily result from sensor motion (hand, whisker, or eye). In contrast, auditory receptors are sensitive to the fundamental dynamic fluctuations of audible acoustic waves. Outlining the core difference between these modalities: acoustic signals always activate the inner-ear haircells, whereas physical objects activate mechano- or photo-receptors primarily upon sensor motion.

Indeed, touch and vision have more in common when interpreting the activation of their respective receptors and when analyzing the operation of their motor-sensory loops. Yet, they also share an important aspect with the auditory sense – in all senses, the frequencies dominating sensory acquisition are in the theta-alpha range, frequencies below 20 Hz. In vision this is the dominant frequency range of ocular drifts during individual fixational pauses. In active touch this frequency range characterizes palpation-induced modulations of mechanoreceptors. In vibrissal active touch it characterizes whisking-induced afferent modulations. In audition it characterizes the syllable-induced modulations of speech signals.

Olfaction and taste are probably as active as touch and vision^105–108^. Hence, their afferent activity is expected to be strongly modulated at the frequencies of sensor motion, frequencies that are similar to (and often synchronized with) those of other facial senses^105,109^.

Thus, it seems that all senses can benefit from a mechanism that locks to the temporal envelope and decodes the information it carries. First, time locking to the low-frequency envelope signal allows a proper decoding of the information that is carried by the envelope itself – speech rate, whisking frequency, or hand/eye scanning speed; the latter are crucial for the control of self-motion. Second, it allows a proper decoding of the information carried by the high-frequency signals modulated by the envelope – syllabic spectral information in speech and texture or shape spatial information in touch and vision. The processing of the high-frequency signals can also benefit from thalamocortical NPLLs that are tuned to the appropriate frequencies^20,62^.

### “Higher-order” thalamic nuclei

An interesting hypothesis suggests that the thalamus is a multi-way relay station, and divides the thalamic nuclei into first- and higher-order nuclei. This division is based on the relative strengths of their peripheral and cortical drives. Nuclei in which peripheral drives are significantly stronger than the cortical drives are termed first-order nuclei; these nuclei (VPM, LGN and MGV) are considered primarily sensory relay stations. In contrast, the nuclei whose cortical drives are considered significantly stronger than their peripheral drives are termed higher-order nuclei, and are regarded as cortico-cortical relay stations. According to this scheme, the thalamic nuclei are all relay stations, but not all of them necessarily relay information between the periphery and the cortex. Rather, higher-order nuclei, such as the POm, MGD, or pulvinar, facilitate communication between various cortical stations^18,110,111^. Importantly, given the poor spatiotemporal resolution of the neuronal responses in these higher-order nuclei, the nature of the information that is supposed to be relayed via cortico-thalamo-cortical pathways is likely only contextual^17,99^.

An alternative hypothesis for thalamic function, the closed-loop hypothesis, assumes that the tight connectivity between thalamus and cortex reflects the fact that thalamo-cortical circuits form processing units. Anatomy indicates that neurons in the granular and sub-granular layers of sensory cortices form anatomical loops with thalamic neurons. For example, layer 4 neurons in S1 of the rat affect the activity of layer 6 neurons, which in turn affect the activity of thalamocortical neurons in the VPM, which then drive layer 4 neurons. Similarly, layer 5a neurons affect layer 5b neurons, which in turn drive POm neurons, which then drive layer 5a neurons (reviewed in ref. ^1,11,20,35^). Similar closed loops (i.e., circuits in which every signal constantly, though not exclusively, affects its source or sources) occur in the visual and auditory systems. As neuronal processing is often iterative, these closed loops can serve as a means by which thalamocortical networks converge upon reliable internal representations^54,112^.

The major debate is thus about the function of the so-called higher-order thalamic nuclei – POm, MGD, and pulvinar. Do they function as cortico-cortical links, or do they process basic sensory information? While this debate is still open, we would like to mention the factors that support the latter view. (i) the so-called higher-order nuclei belong to afferent pathways that, based on comparative anatomy, evolved earlier than those containing the so-called first-order nuclei. Thus, assuming that “higher-order” nuclei function as cortico-cortical links between cortical stations that receive their inputs from “first-order” nuclei raises the question – what were their functions before the so-called first-order pathways evolved? (ii) In contrast, the processing suggested for the so-called higher-order nuclei by the closed-loop hypothesis is consistent with the assumed evolutionary order - processing basic sensory information (sensor motion in touch and vision and temporal envelopes of acoustic waves in audition) that was most likely relevant to animal behavior before the newer pathways evolved. (iii) Processing of these basic signals is essential also for integrating the information arriving from the newer pathways: high-resolution spatial (touch and vision) and spectral (audition) information. Without accurate tracking of sensor motion by central circuits, the high-resolution signals would be meaningless. (iv) The so-called higher-order nuclei receive substantial bottom-up input from the sensory organs, which is at odds with the function of high-order cortico-cortical link. (v) Neuronal populations at the POm function as AND-gates (comparators, as those required for NPLLs), comparing the timing of cortical and sensory inputs^52^, which is again at odds with a cortico-cortical link but consistent with temporal-to-rate recoding.

This debate may be resolved with the aid of systematic investigations of the so-called higher-order nuclei. For example, systematic studies of the POm showed that the nucleus is a patchy structure, containing multiple zones with different morphology and different neuronal response patterns^52,53^; future experiments may test whether different zones take part in different mechanisms. It would be interesting to examine the results of similar studies also in the MGV, where basic systematic thalamocortical studies are still lacking, and in the pulvinar. In any event, the list of counter evidence listed above calls for a reconsideration of the terms “first-order” and “higher-order” with regard to these thalamic nuclei.

Precise manipulation techniques, such as modern optogenetic tools, allow cell-type selective and temporaly precise manipulations of specific cell types in specific brain areas^113,114^ and can thus facilitate such systematic studies. Applying these methods to specific cells in the thalamus and cortex allow (i) testing the effect of activity perturbations on the perception of fine versus coarse object details – the former is expected to be impaired when perturbing NPLL-related cells, (ii) induction of virtual touch that is specific to the NPLL predictions, such as activation of so-called Touch Neurons^25,115^, neurons that are sensitive selectively to touch and not to whisking in air, at times corresponding to the reading of the whisker angle from the rate-coded signals in thalamus or cortex (S1), (iii) virtual contacts can also be induced by abrupt modifications of the rate-coded signals – perturbations like that are expected to be interpreted by the brain as the existence of an external obstacle, (iv) modifying whisking frequency by modulating the population rate-coded signals in cortex or thalamus – such modulations are expected to modify the reading of whisking frequency, thus inducing a correction^55^, (v) modifying the frequency of cortical RCOs by direct optogenetic stimulations should attract the whisking frequency in the direction of the newly-generated cortical frequency, and (vi) inhibiting specific synapses^116^ of the NPLL circuit, such as the inhibitory synapses impinging on POm projecting S1 neurons, should prevent adaptive control of whisking frequency (e.g., in windy conditions^117^).

### Code translation

Neural circuits use a variety of neural codes, depending on their input, output and functional connections. Thus, sensory neurons use temporal and spatial codes that refer to the nature of the objects or waves with which they interact. For example, the temporal frequency of auditory afferents encodes both the spectral frequency of the acoustic wave (up to a certain frequency) and the syllabic rate of a speaker, depending on the applied time windows, or filtering^28^. In vision, depending on the time windows, the temporal frequency encodes object’s texture or motion or eye motion, and temporal phases encode object’s shape^62^. Temporal coding in touch resembles that in vision, yet with modality-specific differences.

Thus, integrating sensory data from different modalities is not trivial. Even within the same modality, neural circuits have to integrate sensory data that are encoded differently, such as data encoded in time (e.g., spatial details) and data encoded in firing rate (e.g., signal intensity). Thus, the brain could use mechanisms for code translation very often^56,118^. NPLLs are one such mechanism. They translate temporal code to population rate code. Judging from the accumulated data, it seems that population rate coding is the code used for cross-modal integration. Moreover, it seems to fit cortical coding of motion control^119–121^, and is thus a suitable code for cross-modal integrative coding.

As mentioned above, optogenetics allows the testing of specific predictions of the NPLL model. As NPLLs recode the information acquired during continuous touching, viewing or hearing, it is not expected to be operative in the detection of brief stimuli. Rather, it is expected to be essential for perceiving features of the sensed objects, such as location, shape, texture, or syllabic rate. Thus, optogenetic experiments aimed at testing perceptual detection cannot be used for testing NPLL’s predictions (e.g.;^122–124^, O’Connor et al. designed a localization task, but the mice turned it into a detection task by whisking only in an area where every detection of an object should lead to a “yes” response).

While experiments with optogenetic perturbation of feature perception are still lacking, an interesting insight may be learned from experiments with intra-cortical optogenetic feedback. Prsa et al. used a rate-to-frequency transformation as a feedback for activating S1 neurons by M1 activity^125^. This fits the frequency-to-rate transformation predicted for S1 neurons by the NPLL algorithm. Prsa et al. used stimulation frequencies <15 Hz, which match the typical whisking frequencies in mice^126^. Similar frequencies were also shown to convey optogenetically-induced rate-coded information between S1 and M1 in mice^127^.

It was recently shown that perceptual phenomena can be substituted, or amplified, by optogenetic stimulation of cortical neurons, in rodents and primates^128,129^. Such experiments suggest that NPLLs are not per se an exclusive operative encoding mechanism, nor a necessary amplification mechanism. Instead, NPLLs are proposed here to be specific translators between temporal and rate coding schemes. As such, NPLLs become crucial computational components during active sensing, when sensory information is temporally encoded. The oscillatory basis of the NPLL mechanism is consistent with studies in which oscillatory cortical stimulations induce phase-specific perceptual enhancements and suppressions^130–133^.

## Summary and conclusions

The role(s) of TC loops has been a subject of several hypotheses over the years. TC loops were hypothesized to function as tunable oscillators, attentional filters, switchable relay stations, cortico-cortical shortcut pathways, or neuronal phase-locked loops transforming temporal- to rate-codes. We argue that the empirical data collected so far about TC loops across senses does not leave too many options regarding their functions. The tight feedback connections almost dictate repetitive processing between the thalamus and the cortex. In addition, the inclusion of oscillating cells in such loops strongly suggests that this processing relates to timing. Based on the similarity between senses, the consistency of thalamocortical phase-locked loop processing with empirical data and evolutionary traces, and the inconsistency of the major alternative hypothesis with evolutionary traces and anatomy, we propose that the mammalian TC systems include loops that translate temporal-coding to rate-coding via neuronal phase-locked loop processing.

If this is indeed the case, then it implies that the common coding scheme in cortical networks is rate coding, and that part of the TC loops translate the information collected with moving sensors into a population rate-coding scheme. An important component of this code translation is temporal predictive processing; this translation mechanism is based on a comparison of the actual timings of sensory inputs against those predicted by the relevant cortex, by virtue of its adaptive local oscillations.

Proposing a unified mechanism to three different sensory modalities is presumptious. Inspired by the tactile system, where evidence is compelling, we suggest here a similar framework for the other senses, which have similar functional requirements, anatomical structure and physiological properties. Thus, the current paper should be taken as proposing a common theoretical formulation that provides testable predictions for future research.

### Reporting summary

Further information on research design is available in the Nature Portfolio Reporting Summary linked to this article.

## Supplementary information

Reporting Summary
